# The Roles of Exosomal Proteins: Classification, Function, and Applications

**DOI:** 10.3390/ijms24043061

**Published:** 2023-02-04

**Authors:** Xin-Xin Li, Lu-Xuan Yang, Chuang Wang, Hui Li, De-Shun Shi, Jian Wang

**Affiliations:** State Key Laboratory for Conservation and Utilization of Subtropical Agro-Bioresources, College of Animal Science and Technology, Guangxi University, Nanning 530004, China

**Keywords:** exosome, proteins, disease, biomarker, targeted delivery

## Abstract

Exosome, a subpopulation of extracellular vesicles, plays diverse roles in various biological processes. As one of the most abundant components of exosomes, exosomal proteins have been revealed to participate in the development of many diseases, such as carcinoma, sarcoma, melanoma, neurological disorders, immune responses, cardiovascular diseases, and infection. Thus, understanding the functions and mechanisms of exosomal proteins potentially assists clinical diagnosis and targeted delivery of therapies. However, current knowledge about the function and application of exosomal proteins is still limited. In this review, we summarize the classification of exosomal proteins, and the roles of exosomal proteins in exosome biogenesis and disease development, as well as in the clinical applications.

## 1. Introduction

In recent years, extracellular vesicles (EVs), lipid bilayer membrane vesicles with diameters ranging from nano- to micro-meters, have received increasing attention as critical mediators of cell-to-cell communication [1,2,3,4,5]. EVs have been classified into several categories: exosomes (30–150 nm), ectosomes (50–10,000 nm), migrasomes (500–3000 nm), exosomeres (<500 nm), exophers (1000–10,000 nm), apoptotic bodies (50–5000 nm) and so on [6]. All cells are able to release EVs with different sizes, contents, and concentrations according to the purpose, cell types, and cell conditions [4]. Given their diverse sources, EVs exist in almost all biological fluids and are associated with extensive physiological processes, such as cancer progression, immune responses, neurological disorders, cardiovascular diseases, and viral pathogenicity [4,7,8]. As a result, they have considerable potential utility in disease diagnosis and targeted delivery [4,9,10,11].

As a subpopulation of EVs, exosomes originate from endosomes which are generated from the budding of the invagination of the membrane of a multivesicular body (MVB), and are released into the extracellular space upon the fusion of multivesicular endosomes (MVEs) with the cell membrane [12]. Exosomes have become a focal point of biomedical research given their highly diverse cargoes (nucleic acids, proteins, lipids, and metabolites, etc.). This interest is due to their broad roles in regulating biological processes and their great potential for clinical applications [4,13,14,15,16]. In addition to exosomal non-coding RNAs (ncRNAs), exosomal proteins have received particular attention because of their wide distribution (from the membrane surface to the interior of exosomes), various types, and diverse functions [17]. This review discusses the recent studies exploring exosomal proteins’ biological functions and potential applications.

## 2. Classification of Exosomal Proteins

Exosomal proteins are essential elements of exosomes and are distributed from the exosomal membrane surface to the interior. To date, ~41,860 exosome-associated proteins among 10 analyzed species have been identified based on the Exocarta database (http://www.exocarta.org (accessed on 15 December 2022)) [18].

**Figure 1:**
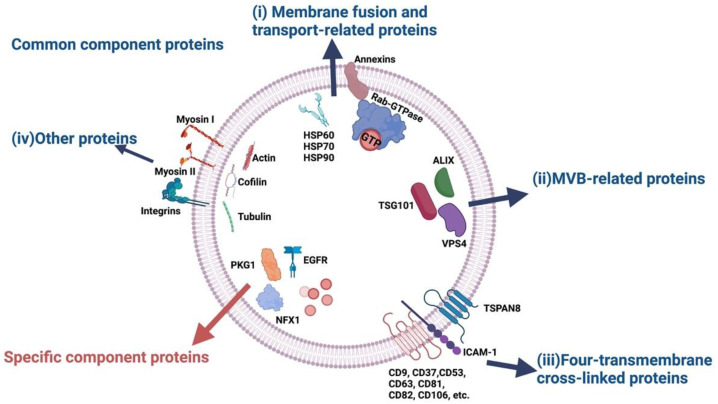
Common component proteins and specific component proteins. Common component proteins include: (i) membrane fusion and transport-related proteins, such as Rab-GTPases, annexin, HSP60, HSP70, and HSP90; (ii) MVB-related proteins, such as ALIX, TSG101, and VPS4; (iii) four-transmembrane cross-linked proteins, such as ICAM-1, TSPAN8, CD106, CD82, CD81, CD63, CD53, CD37, and CD9; and (iv) other proteins, such as Myosin I, Myosin II, and Myosin III. Specific component proteins, such as PKG1, EGFR, and NFX1, exist in specific cells, which depends on the types and conditions of the parent cells.

There is no standard for classifying exosomal proteins, and they can be roughly divided into two categories: Common component proteins and specific component proteins [19] (Figure 1). Common component proteins contain four main sub-categories: (i) membrane fusion and transport-related proteins, including Ras-associated binding (Rab)-GTPases, annexin, and heat shock proteins (HSPs) such as HSP60, HSP70, and HSP90; (ii) MVB-related proteins (also called endosomal-sorting complex that is required for transport [ESCRT] complex related proteins), including tumor ALG-2-interacting protein X (ALIX), susceptibility gene 101 (TSG101), and vacuolar protein sorting-associated protein 4 (VPS4); (iii) four-transmembrane cross-linked proteins, such as intercellular adhesion molecule 1 (ICAM-1), tetraspanin-8 (TSPAN8), CD106, CD82, CD81, CD63, CD53, CD37, and CD9; and (iv) other proteins, including integrins, actin, myosin, cofilin, tubulin, and others, that are common components of exosomes and participate in their basic construction of exosomes, such as cytoskeletal construction [19,20].

The second category includes the specific component proteins that depend on the types and conditions of the parent cells of the exosome. For instance, cGMP-dependent protein kinase 1 (PKG1) and x-box-binding protein 1 (NFX1) are only detected in plasma exosomes [21]. Meanwhile, epidermal growth factor receptor (EGFR) has significantly high expression levels on the surface of lung cancer cell-secreted exosomes [22].

## 3. The Functions of Exosomal Proteins in Exosome Biogenesis

### 3.1. Exosomal Proteins in the ESCRT-Dependent Pathway

**Figure 2:**
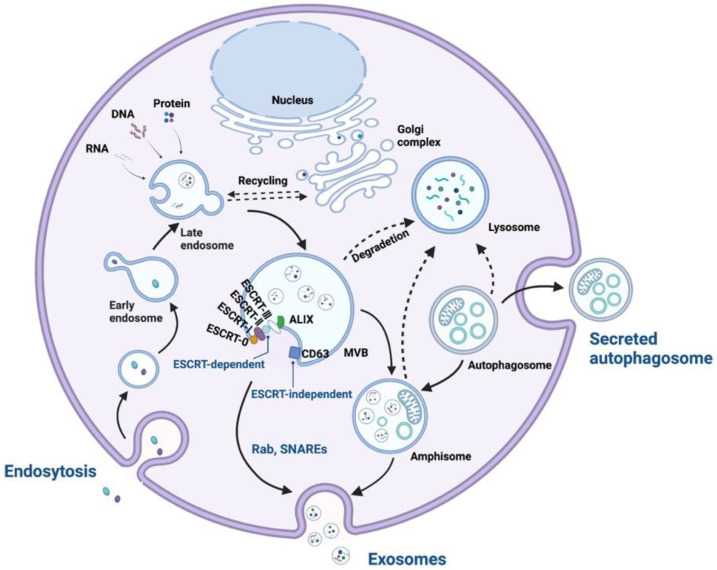
The overview of exosome biogenesis and related proteins. First, the cell plasma membrane buds to form an early endosome. Then, the early endosomes give rise to late endosomes. Usually, multiple late endosomes are packaged into MVBs. MVBs are the center of exosome biogenesis and can communicate with organelles, such as Golgi and endoplasmic reticulum to exchange cargo. In ESCRT-dependent pathway, TSG101, VPS4, and ALIX participate in forming ESCRT-complex and releasing exosomes from MVBs. In ESCRT-independent pathway, CD63 can help MVB release exosomes. Other proteins, including Rab and SNAREs, are responsible for membrane fusion and releasing exosomes outside cells.

Exosome biogenesis is a highly regulated process in which exosomal proteins play several roles. Exosomes originate from endosomes that form from the invagination of the plasma membrane. The process from early endosomes to multivesicular bodies (MVBs) requires the formation of ESCRT machinery in the ESCRT-dependent exosome pathway [4] (Figure 2). ESCRT consists of four complexes (ESCRT-0, -I, -II, and -III) and associated proteins, including TSG101, VPS4, and ALIX [23]. TSG101 is an element of the ESCRT-I complex, which is responsible for the release of exosomes. TSG101 recognizes the short linear motif: P(T/S)AP through the UEV protein domain of the VPS23/TSG101 subunit. This allows reverse-topology budding and the production of multivesicular bodies (MVBs), which transport degradable cargoes to lysosomes [24]. VPS4 is a member of the ESCRT-III complex and it fuels ESCRT-dependent membrane fission reactions [25]. As for ALIX, it can enroll ESCRT-III proteins in endosomes [26]. ALIX is composed of a BRO1 domain at its N-terminus, a V-shaped domain in the middle, and a proline-rich, flexible region at its C-terminus (PRR) [27]. Both the BRO1 domain and the PRR bind to tumor susceptibility gene 101 protein (TSG101) in ESCRT-I [28,29], and the BRO1 domain can also interact with charged multivesicular body protein 4 (CHMP4) [29,30,31,32,33]. G protein–coupled receptors PAR1 and P2Y1 contain a YPX3L motif in their cytosolic regions which, together with the V-domain, binds ubiquitin [34].

### 3.2. Exosomal Proteins in the ESCRT-Independent Pathway

Apart from the ESCRT-dependent exosome pathway, emerging studies have pointed to an ESCRT-independent pathway that is involved in the biogenesis of exosomes and in loading their cargoes with the help of exosomal proteins [35] (Figure 2). For example, CD63, a member of the tetraspanin family (TSPAN), is abundant in late endosomes and lysosomes in a variety of cell types. It acts as a critical effector of an ESCRT-independent MVE-generating process that is eventually needed to commence the polymerization of PMEL lumenal domains into functional amyloid fibrils [36,37].

### 3.3. Exosomal Proteins in Membrane Fusion and Exosome Release

Usually, after the formation of MVBs, exosomes are released after the MVBs fuse with the cellular plasma membrane. This process requires the participation of Rab-GTPases, tethers, and soluble N-ethylmaleimide-sensitive factor attachment proteins receptors (SNAREs) [38,39,40,41] (Figure 2). Rab-GTPs can bind to tethering factors; for example, Rab5 can enhance the generation of the PI3 phosphate (PI3P), which functions as a binding partner of many endosomal effectors by binding with PI3-kinase Vps34 [42]. In addition, other Rabs, such as Rab11 and Rab4, play a role in recycling events by forming distinct domains [43,44].

SNAREs play a vital role in intracellular membrane fusion. Typically, SNAREs are divided into t-SNAREs and v-SNAREs [45]; the former are located on the destination membrane, while the latter are present on the transport vesicles [46,47]. Previous studies show that v-SNAREs, such as vesicle-associated membrane protein 7 (VAMP7) and SEC22 homolog B, and vesicle trafficking protein (SEC22B) are necessary for the unconventional secretion of extracellular vesicles [48,49]. In addition, ER-derived vesicles and mitochondria-derived vesicles can fuse with VAMP7+ late endosomes/MVBs through a process that is SNARE-dependent and involves VAMP7, Stx5, and SNAP47 [48]. VAMP7, t-SNARES Syntaxin4, and SNAP23 are all required for the process of lysosomal exocytosis (the unusual secretion of lysosomal contents upon the fusion of lysosomes with the plasma membrane). Finally, together with SNAP23 or SNAP29 and PM SNAREs Syntaxin3/4, SEC22B has been found to have a role in the unconventional secretion of leaderless proteins such as IL-1 and ferritin [49,50].

Proteins participate in almost every step of exosome production, including endocytosis, exosome release, and interaction with recipient cells. The ESCRT-dependent pathway is still the most important way to produce exosomes, and the ESCRT-complex is protein-based. Proteins also participate in the ESCRT-independent pathway; one example is CD63. Without Rab-GTPases and SNAREs, exosomes cannot be released. Though the roles of proteins in biogenesis are not yet fully understood, studies have confirmed the importance of exosomal proteins; still, further in-depth exploration is required.

## 4. The Roles of Exosomal Proteins in Disease Development

Exosomal proteins modulate various processes involved in multiple diseases, such as the formation of cancer microenvironments, tumor progression, tumor metastasis, infection, and nervous system diseases, among others [4,51,52]. The roles of exosomal proteins differ between diseases and should be analyzed in the context of a particular condition. In this section, we will examine the roles of exosomal proteins in carcinoma, sarcoma, melanoma, neurological disorders, immune responses, and infection.

### 4.1. Exosomal Proteins and Carcinoma

Exosomal proteins influence the development of carcinoma, which is the most common type of cancer and mainly affects organs and glands, including breasts, ovaries, lungs, the pancreas, and others [53,54,55,56]. Epidermal growth factor receptor (EGFR) has been identified in the exosomes of multiple cancer cells, including lung carcinoma cells, colorectal carcinoma cells, and skin epidermoid carcinoma cells [57]. EGFR acts as an essential regulator in cancer metastasis. For instance, gastric cancer cell-derived exosomal EGFR can be transported to the liver and then integrated into the plasma membrane of hepatic stromal cells [58]. In recipient hepatic stromal cells, exosomal EGFR can efficiently activate hepatocyte growth factor (HGF) by inhibiting miR-26a/b levels [58]. The increase in paracrine HGF, which binds the c-MET receptor on the migrated cancer cells, contributes to the formation of a microenvironment that allows metastatic cancer cells to land and proliferate in liver tissue [58]. Additionally, exosomal EGFR can activate the AKT and MAPK signaling pathway to human umbilical vein endothelial cells (HUVECs) by conveying cancer cell-derived EGFR signals, which is accomplished via the presentation of intact EGFR kinase activity and phosphatidylserine (PS) [57,59]. Moreover, oncogenic EGFR signaling might be responsible for two processes that occur simultaneously in HUVECs: the generation of vascular endothelial growth factor (VEGF) and the autocrine signaling of VEGF receptor-2 (VEGFR-2) [59]. In addition, tumor xenograft research in a mouse model revealed that tumor-derived exosomes may be able to transfer EGFR to tumor blood vessels, which may then promote the tumors’ development and angiogenesis [60]. Exosomal EGFR levels in the plasma of xenograft mice exhibited the same trend as the growth of xenograft tumors, suggesting that these levels could be used to assess tumor size [60]. In addition, the levels of exosomal EGFR levels were significantly higher in five out of nine lung cancer patients when compared to healthy individuals while the levels of soluble EGFR in plasma were not significantly different in seven out of nine lung cancer patients [60]. Moreover, exosomal EGFR has been detected in fresh lung biopsies of non-small cell lung cancer (NSCLC) patients [61]. EGFR was also shown to be positive in over 80% of exosomes from NSCLC patients, while less than 2% of chronic lung inflammation was discovered to be EGFR positive; this suggests that EFGR could serve as an indicator to distinguish between NSCLC and chronic lung inflammation [59].

Exosomal proteins are also a crucial contributor to drug resistance during cancer treatment. For example, they can mediate chemotherapeutic drug export to cause drug resistance during breast cancer treatment, one of the most prevalent malignancies in women [62]. In one study, mitoxantrone-resistant breast cancer cells were able to secrete ATP binding cassette subfamily G member 2 (junior blood group) (ABCG2) proteins to exosomes to affect the neighboring breast cancer cells, inducing multidrug resistance [63,64]. Moreover, exosomal proteins are associated with chemoresistance in ovarian cancer, another common lethal gynecological cancer [65]. A recent study indicated that plasma gelsolin (pGSN), an isoform of the GSN protein secreted by chemoresistant ovarian cancer cells, was delivered into exosomes and activated α5β1 integrin. This resulted in an increase in hypoxia-inducible factor 1 subunit alpha (HIF1α), which is associated with ovarian cancer tumor development. Consequently, chemo-sensitive cells transformed into resistant counterparts, promoting ovarian cancer cell chemoresistance and survival [65,66,67,68].

### 4.2. Exosomal Proteins with Sarcoma

Exosomal proteins are important in the development of sarcoma, which usually occurs in soft or connective tissues, such as muscle, fat, tendons, blood vessels, cartilage, ligaments [69]. Osteosarcoma, the most common primary malignant bone tumor, has a high propensity to spread to other parts of the body with the assistance of exosomal proteins [70]. A comparison of the protein profiles between osteosarcoma cancer cell-derived exosomes and exosome-free media demonstrated significant differences [71]. A study confirmed that osteosarcoma-derived exosomal proteins promote metastasis [72]. In this case, plasma exosomes in 70 osteosarcoma patients, 22 healthy individuals, and nine patients with benign tumors were examined, and the results implied that osteosarcoma patients had a higher expression of exosomal PD-L1 when compared with healthy individuals [72]. Meanwhile, osteosarcoma patients with lung metastasis also showed a higher PD-L1 expression level in exosomes than those without lung metastasis [72]. Further investigation revealed that exosomal PD-L1 and N-cadherin contributed to the pulmonary metastasis of osteosarcoma, suggesting that plasma exosomal PD-L1 and N-cadherin might serve as predictors of pulmonary metastasis in osteosarcoma patients [72].

Fusion genes are also critical oncogenic contributors to osteosarcoma. For example, osteosarcoma-derived exosomal Rab-22A-NeoF1 fusion protein enhances the establishment of the pulmonary pre-metastatic niche by recruiting bone marrow-derived macrophages [73]. Exosomal proline-rich tyrosine kinase 2 (PYK2), a binding protein of Rab-22A-NeoF1, is released together with Rab-22A-NeoF1 with the assistance of HSP90 through a KFERQ-like motif. This leads to the activation of ras homolog family member A (RHOA) in its negative recipient osteosarcoma cells, as well as transcription 3 in its recipient macrophages, therefore increasing the M2 phenotype [73].

### 4.3. Exosomal Proteins and Melanoma

Several proteins have been identified in melanoma-derived exosomes, and many of them are related to melanoma metastasis and progression. Compared to normal melanocytes exosomes, melanoma-derived exosomes contained more proteins. These include melanoma-specific proteins, such as an isoform of HSP70, which is melanoma-specific [74,75]. Other proteins are also enriched in malignant melanoma cell-derived exosomes, indicating their importance to melanoma development; these include tyrosinase related protein 2 (TYRP2), HSP70, melanoma antigen recognized by T-cells 1 (MART1), major histocompatibility complex (MHC I), annexin A2, GTP-binding proteins, human epidermal growth factor receptor 2 (Her2/neu), CD44, GTP-binding proteins, and premelanosome protein (PMEL) [76,77,78]. Moreover, melanoma cells that expressed Rab27a generated exosomes that were packed with many proteins connected to melanoma development [79]. As soon as these exosomes were taken up by Rab27a-low melanoma cells, these cells’ invasion potential was enhanced [79]. Furthermore, a reduction in metastasis in vivo was observed after the knockdown of *Rab27a* in melanoma mouse models, verifying Rab27a’s role in pro-metastatic alterations [79].

Annexin A2 is another representative protein with a connection to melanoma development and progression. This protein is present on the exosome membrane surface and can combine with S100 (which is known to locate on the cell surface) to form a tetramer. This enhances ceramide-1-phosphate–dependent vascular endothelial cell invasion, which is thought to allow melanoma cells to hijack the exosomal transport of annexins to promote invasion [80,81,82,83,84].

### 4.4. Exosomal Proteins and Neurological Disorders

Exosomal proteins play complex and diverse roles in the development of neurological diseases. Alzheimer’s disease (AD) is a typical debilitating neurological disease with the primary pathology hallmarks of the cumulation of Aβ plaque and neurofibrillary tangles (NFTs), which are generated by the hyperphosphorylation of Tau protein [85]. Studies have revealed that exosomes carry Aβ peptide and Tau protein, affecting the development of AD [85,86,87]. For example, proteomic analysis of exosomes suggested that the exosomal protein profile of human iPSC neurons that expressed a type of mutant Tau protein (mTau) differed from that of normal human iPSC neurons, including the presence of PP2A phosphatase inhibitor. This indicates that exosomal mTau with other cargoes might affect the spread of phosphorylated Tau as well as the development of AD [86]. Additionally, earlier investigations showed that the amyloid precursor protein (APP), amyloid intracellular domain, and APP-C-terminal fragment are all released by the exosomes of differentiated neuroblastoma cells and primary neuronal culture cells [88].

Apart from exosomes’ and exosomal proteins’ roles in promoting AD development, many researchers have also demonstrated the beneficial actions of exosomal proteins in AD. In particular, exosomal cystatin C is thought to have multiple strong neuroprotective effects [89]. Mouse primary neuron-derived exosomes included at least nine distinct O-glycoforms of cystatin C, and their levels decreased in mutant PSEN2–overexpressed cells [89]. Finally, neprilysin, a vital Aβ-degrading enzyme in the human brain, was found in adipose tissue–derived mesenchymal stem cells, indicating that exosomal neprilysin might participate in Aβ degradation in the human brain [89].

Exosomal proteins also have connections with the development of Parkinson’s disease (PD), which is another typical debilitating neurological disorder. Its significant hallmarks include the accumulation of misfolded alpha-synuclein (α-syn), which leads to the advanced loss or even demise of dopaminergic neurons in the striatum and substantia nigra [90,91]. Transmission between cells is a key role of exosomal proteins in PD, especially the transmission of exosomal α-syn [92]. Although exosomes contain modest quantities of α-syn, studies have shown that they create an optimal environment for α-syn to assemble, which could contribute to the progression of PD pathology [93]. Compared with free α-syn, the exosomal α-syn is more able to promote PD development, as it is more easily taken up by recipient cells [94]. Exosomal proteins that are secreted by immune cells, such as dendritic cells and B lymphocytes, also mediate PD development; the activation of the peripheral immune system enhances the discordant central inflammatory response and synergistically driving neurodegeneration [95]. Dendritic cell-derived exosomal MHC class II promotes T-cell activation by combining with the antigenic peptide [96]. Moreover, the presence of MHC class I and II antigens in dendritic cell exosomal membranes, as well as costimulatory and adhesion molecules (such as CD80, CD86, and CD40), all contribute to the propagation of the inflammatory response [97].

Other exosomal proteins have been shown to affect the development of other neurological disorders. For instance, exosomal superoxide dismutase 1 (SOD1), exosomal dipeptide-repeat proteins (DPRs), exosomal TAR DNA-binding protein (TARDBP), and fused in sarcoma (FUS) have all been found to affect the spread of amyotrophic lateral sclerosis [98,99,100,101,102]. Although emerging studies have revealed that exosomal proteins contribute to the development of neurological disorders, the specific mechanisms remain far from clear.

### 4.5. Exosomal Proteins in Immune Responses and Infection

Exosomal proteins have also been studied extensively for their potential roles in immunological responses. For instance, programmed death such as 1 (PD-L1), a membrane-bound ligand located on the surface of sundry types of cells, was reported to increase in the presence of inflammation and/or a variety of oncogenic diseases [103]. It inhibits antigen-driven T-cell activation by binding to the PD-1 receptor on immunological T-cells, which triggers the dephosphorylation of the T-cell receptor and its co-receptor CD28; this dephosphorylation is driven by the src homology region 2 (SH2)–containing protein tyrosine phosphatase 2 (SHP2) [104]. It was previously believed that PD-L1 mainly functioned inside the tumor bed, which suggested that it directly interacted with PD-1 on the surface of tumor-infiltrating lymphocytes [105]. However, a recent study suggested that cancer cells released the overwhelming majority of their PD-L1 through exosomes rather than presenting it on the surface of their cells. Moreover, exosomal PD-L1 acted as a suppressor of the antitumor immune response, inducing systemic antitumor immunity and memory [106]. By knocking out *Rab27a* and *nSMNase2*, two important exosomal biogenesis-related genes, exosomal PD-L1 was proven to suppress T-cell activity to promote multiple tumor growth, including prostate cancer and melanoma. Meanwhile, exogenously introduced exosomal PD-L1 was able to rescue immune suppression and tumor growth [106]. This study suggested exosomal PD-L1 as a potential therapeutic target to circumvent resistance to existing antibody-based methods [106]. Another example showed that glioblastoma-derived exosomal CD152 could suppress the immune response [107]. Specifically, in CD8+ T-cells, exosomal CD152 participated in the inhibition of TNF-α and INF-γ, which release and mediate cell apoptosis, as well as CD4+ T-cell activation, inhibiting the immune response and promoting tumor progression [107].

Exosomal proteins also have a connection with infectious agents related to immune responses, including bacteria, fungi, viruses, and parasites [108,109]. On the one hand, some cells infected with pathogenic microorganisms secrete proteins via exosomes to promote disease development. For example, the exosomal membrane proteins CD9 and CD81, were observed to enhance the HIV access into cells [110]. Similarly, the exosomal phosphatidylserine (PtdSer) receptor T-cell immunoglobulin and mucin domain-containing 4 (TIM-4) may facilitate the cellular entry of human immunodeficiency virus 1 (HIV-1) due to its PtdSer-rich envelope [111].

On the other hand, exosomal proteins can suppress pathogen infection [112]. For example, exosomal apolipoprotein B mRNA editing enzyme catalytic subunit 3g (A3G), derived from naïve macrophages, decreased the accumulation of products from HIV-1 reverse transcription as well as steady-state levels of HIV-1 Gag and viral infectivity factor (VIF) proteins, thus providing uninfected T-cells with resistance to sustained HIV-1 proliferation [113].

### 4.6. Exosomal Proteins in Cardiovascular Diseases

Exosomal proteins play important roles in cardiovascular disease and cardioprotection. Many efforts have been made to explore the cardiovascular exosomes and proteins. For instance, arrhythmogenic cardiomyopathy is an inherited heart disease and immortalized cardiosphere-derived cells expressing high levels of β-catenin are therapeutically potent to arrhythmogenic cardiomyopathy and exosome proteinomics demonstrated that differentially expressed proteins or unique to immortalized cardiosphere-derived cells are chaperone proteins, metabolite interconversion enzymes, and cytoskeletal proteins between primary cardiosphere-derived cells and immortalized cardiosphere-derived cells, suggesting the potential participation of exosomal proteins in arrhythmogenic cardiomyopathy therapy [114]. Moreover, highly differentiated adult cardiomyocytes were able to release exosomal HSP60 under hypoxic stress, which might be detrimental to the surrounding cardiomyocytes as HSP60 could lead to cardiomyocyte apoptosis by activating Toll-like receptor [115,116]. In cardioprotection, proteinomics analysis of exosomes has revealed 22 and 12 exosomal proteins which were pro-survival in the plasma of cardiac surgery patients 3h after aortic unclamping and coronary artery bypass graft patients, respectively [117]. This indicated the potential of exosomal protein-based protection for aged hearts. Another study has indicated the encouraging potentiality of cardiac-resident progenitor cells-derived exosome and exosomal proteins for myocardial infarction. In particular, exosomal pregnancy-associated plasma protein-A (PAPP-A), could cleave IGF binding proteins to release insulin-like growth factor-1 (IGF-1) which is a key cardioprotective factor [118,119,120]. Selective PAPP-A knockdown prevented the cardioprotective activities of cardiac-resident progenitor cell-derived exosomes both in vitro and in vivo [120].

## 5. Applications of Exosomal Proteins

### 5.1. Exosomal Proteins as Biomarkers

Early diagnosis is one of the most important steps in clinical practice, as it opens the door to future care and treatment. Exosomal proteins have garnered much interest as biomarkers in recent years since they are the common component of exosomes and can reflect both the physiological and pathological states of parent cells [121,122,123,124].

Exosomal proteins have many advantages for disease diagnosis. First, proteins in exosomes are stable; they have a long half-life because the exosome membrane protects them from free proteases in body fluids [125]. For example, phosphorylated proteins can remain stable in exosomes for as long as 5 years at −80 °C [126]. Second, exosomal proteins can exert their effects immediately in recipient cells, while exosomal DNAs/mRNAs must be transcribed/translated before they have any effect [125]. Consequently, quantitative and qualitative data on exosomal proteins may give more precise information than data on other cargoes [125]. Third, in comparison to other exosome cargoes, exosomal surface proteins can be identified in a smaller amount of sample material after an isolation technique that is comparatively straightforward, making such proteins easy to detect [127].

Exosomal proteins are considered a diagnostic indicator of various malignancies. In general, cancer cells can express abnormal levels of proteins that can be packaged in exosomes. For example, a study including 190 pancreatic ductal adenocarcinoma (PDAC) patients demonstrated that glypican-1 (GPC-1) was markedly higher in the patients’ serum exosomes than in healthy individuals. Further exploration revealed that at least part of the exosomal GPC-1 was produced by PDAC tumors given the presence of a mutant transcript of kirsten rat sarcoma virus (*Kras*), the oncogenic gene that is commonly seen in PDAC. This suggests that exosomal GPC-1 has potential as a biomarker for pancreatic cancer [128]. Meanwhile, survivin-2B, an inhibitor of cancer cell apoptosis, is significantly overexpressed in breast cancer patient sera exosomes, demonstrating that sera exosomal surviving-2B may be a potential breast cancer biomarker [129]. Finally, a proteomic analysis identified 2964 proteins in the urine exosomes of 28 bladder cancer patients, revealing that exosomal trophoblast cell surface antigen 2 (TACSTD2) was significantly higher in the patients’ urine than in individuals with hernias. This finding highlights the potential of TACSTD2 as a biomarker of bladder cancer [130].

Exosomal proteins have also been recommended as biomarkers for other diseases, such as neurodegenerative diseases and urinary system-related diseases. A study including three amyotrophic lateral sclerosis (ALS) male patients (average age 42 ± 3.06 years) and three healthy male controls (average age 39.33 ± 3.79 years) demonstrated that the level of plasma exosomal coronin-1a (CORO1A) of ALS patients was 5.3 times higher in the ALS patients than that of healthy controls. Further exploration revealed that exosomal CORO1A may participate in the onset and progression of ALS by blocking autophagic flux, indicating that it is a candidate biomarker for the disease [131]. Usually, liver-derived Fetuin-A protein is released into the plasma and is not detectable in urine. However, in acute kidney injury (AKI), many molecules, including Fetuin-A, could escape tubular reabsorption due to the filter at the glomerulus. Thus, to detect the existence in urine, Fetuin-A can be used to evaluate AKI and to detect the level of exosomal Fetuin-A in urine [132].

Some examples of exosomal proteins with the potential to be used as disease biomarkers are summarized in Table 1.

### 5.2. Exosomal Proteins with Targeted Delivery

As natural carriers, exosomes can transport internal cargoes to specific recipient cells in a targeted manner. This endows them with great potential as a targeted delivery platform, and exosomal proteins play a crucial role in determining specific targeted delivery. The interactions between tetraspanins and specific transmembrane proteins of recipient cells contribute greatly to the specific recognition [166]. In particular, the preferential interaction between TSPAN8, an abundant protein on the exosomal membrane surface, and α4/β4 integrin chains has received much attention [166]. For example, exosomes containing TSPAN8-α4 complexes are most readily taken up by endothelial and pancreatic cells, with CD54 as the main ligand [166]. In addition, other exosomal proteins also affect delivery. For instance, exosomal CD47 on the membrane could induce a signal that means “don’t eat me”, protecting exosomes from phagocytosis and limiting their clearance from the circulation, therefore facilitating the delivery of their internal contents [167].

Genetic engineering can help guide the fusion of the gene sequence of a protein or peptide with the gene sequence of a selected exosome membrane protein for specific targeting, and this approach is very effective for the surface display of peptides and proteins [168,169]. In general, genetic engineering fuses homing peptides or ligands with exosomal transmembrane proteins using plasmids that encode the fusion proteins, which gives rise to the secretion of exosomes with targeting ligands on the surface [170]. Among the diverse exosomal surface proteins, exosomal lysosome–associated membrane protein-2B (LAMP-2B) is one of the most widely used. LAMP-2B consists of a 25AA signal peptide, where a luminal N-terminus, transmembrane domain, and the C-terminus. Targeting can be achieved by inserting the target peptide/protein in-frame between amino acid residues 39 and 40. The engineered targeting peptide/protein will be displayed on the membrane surface when the fusion cassette is expressed [171]. For example, the fused αγ integrin-specific iRGD peptide (CRGDKGPDC)-LAMP-2B protein on the exosome membrane surface could efficiently target integrin-positive breast cancer cells to deliver doxorubicin (DOX) via intravenous injection [172]. Moreover, a plasmid containing the rabies viral glycoprotein (RVG) peptide (a specific binder to acetylcholine receptors) and LAMP-2B fusion sequences has successfully expressed the fusion protein on the exosome membrane surface after transfection in self-derived dendritic cells, which can deliver siRNAs in vivo and in vitro [171]. Other exosomal proteins, including platelet-derived growth factors (PDGFRs), Lactadherin, CD9, CD63, and CD81, have also been widely used for constructing fusion proteins in exosome targeting [170,173,174,175].

Covalent binding–based chemical modification is another efficient way to modify exosomal proteins for specific targeting. Click chemistry, a covalent binding approach, can be used to modify the amine group of exosomal protein using alkyne groups and a copper-catalyzed azide-alkyne cycloaddition (CuAAC) “click” reaction [176]. In one study, the sulfonyl azide-mediated cycloaddition reaction successfully attached a glioma-targeting RGE peptide to the exosome surface. After penetrating the blood–brain barrier following intravenous administration, the modified RGE exosomes were able to target tumor areas [175]. Nevertheless, although the CuAAC click reaction will not impair the normal function of exosomes, the copper catalyst may be cytotoxic [177].

## 6. Conclusions

As one of exosomes’ most abundant components, exosomal proteins play a significant role as a mediator in intercellular communication, especially in specific recognition among cells. This review has highlighted the classification of exosomal proteins (4 main categories) as well as some of the recent progress on this topic. In particular, recent research has established the roles of exosomal proteins in the development of multiple diseases, including carcinoma, sarcoma, melanoma, neurological disorders, immune responses, infection, and cardiovascular diseases, and the potential applications for diagnostic biomarkers and specific targeted delivery.

Exosome proteomics has evolved as a distinct field to evaluate the roles of exosomal proteins under pathological and physiological conditions and to understand how they participate in pathogenesis. However, challenges remain in studying the mechanisms of exosomal proteins and applying them in practice.

The first challenge concerns storage conditions, which can affect the proteomic content of exosomes; for example, the proteomics signature differs when exosomes are stored at +4 °C or −80 °C. In addition, due to current technological limitations, it is difficult to isolate pure exosomes or specific subtypes of exosomes from a mixture of different vesicle types, which limits the exploration of exosomes’ functions and mechanisms. Therefore, suitable standards and more sophisticated techniques are required to isolate, purify, and characterize exosomes.

Although emerging studies have demonstrated exosomal proteins’ great value as biomarkers for disease, the accuracy of a single exosomal protein might not be enough, which is a major hindrance for clinical applications. To solve this challenge, to combine more exosomal molecules—including other exosome surface markers, exosomal lipids, and exosomal nucleic acids—may be an effective way to improve accuracy.

It remains difficult to achieve precise targeting when applying exosomes as carriers for targeted therapy, and more specific binding between exosomes and various cells still needs to be explored. In addition, the large-scale production of exosomes is essential for clinical applications, but traditional cell culture is unaffordable in terms of cost and time. Therefore, there is an urgent need to develop more economical methods to isolate and purify exosomes. Finally, determining the dose and potency of exosomes for clinical applications remains a challenge; exosomes are heterogeneous among different progenitor cells and animal models used for exosome therapy, and there are differences in cell characteristics and evaluation. Therefore, extrapolating exosome doses from preclinical models to clinical trials requires careful consideration.

## Figures and Tables

**Table 1:** Summary of exosomal proteins as potential biomarkers for diseases.

Biofluid	Related Diseases	Potential Biomarkers	References
Serum	Lung cancer	CD5L	[55]
Serum	Non-small cell lung cancer	PLA2G10	[133]
Serum	Non-small cell lung cancer	Fibronectin	[134]
Serum	lung adenocarcinoma	ITGAM & CLU	[135]
Serum	Non-small cell lung cancer	AHSG & ECM1	[136]
Serum	Prostate cancer	FLNA	[137]
Serum	Amyotrophic lateral sclerosis	TDP-43	[138]
Serum/plasma	Breast cancer	CD82	[139]
Plasma	Early-stage lung adenocarcinoma	GCC2	[140]
Plasma	Non-small cell lung cancer	TLN1, TUBA4A and HSPA8	[141]
Plasma	Small cell lung cancer	F13A1 and CFHR4	[142]
Plasma	lung adenocarcinoma	PDGFA	[143]
Plasma	Non-small cell lung cancer	MUC1	[144]
Plasma	lung adenocarcinoma	FGB and FGG	[145]
Plasma	Parkinson’s disease	CLU, C1R subcomponent and APOA1	[146]
Plasma	Parkinson’s disease	α-syn	[147]
Plasma	Traumatic Brain Injury	Aβ42, P-tau, PrPc and SYNGR3	[148]
Plasma	Colorectal cancer	CPNE3	[149]
Plasma	Cardioprotection	HSP70	[150]
Plasma and CCM	Pancreatic adenocarcinoma	Immunoglobulins	[151]
CCM	Melanoma, non-small cell lung cancer and renal cancer	PD-L1	[106]
CCM	Bladder cancer	TALDO1	[152]
CCM	Breast cancer	Glycoprotein 130	[153]
CCM	Oral squamous cell carcinoma	THBS1	[154]
CCM	Breast cancer	GLUT-1, GPC-1 and ADAM10	[155]
CCM	Pancreatic cancer	ZIP4	[156]
CCM	Colorectal cancer	CA125	[157]
CCM	Acute myocardial infarction	TGS101, HSP70 and CD63	[158]
Urine	Prostate cancer	ACPP, FASN, FKBP5, FAM129A, FOLH1, KLK2, KLK3/PSA, MSMB, NKX3-1, NDRG1, NEFH, RAB27A and TGM4,	[159]
Urine	Prostate cancer	AMBP, CHMP4A, CHMP4C, FABP5 and Granulin	[160]
Urine	Bladder cancer	FOLR1, RAB35, RALB, TMPRSS2 and TPP1	[161]
Urine	Bladder cancer	CEACAM-5, EPS8L2, MSN and MUC1	[162]
Cerebrospinal fluid	Alzheimer’s disease	Tau	[163]
Blood	Alzheimer’s disease	GAP43, NRGN, SNAP25 and SYT1	[164]
Saliva	Inflammatory bowel disease	PSMA7	[165]

CCM: cell culture media; CD5L: CD5 molecule like; GCC2: GRIP and coiled-coil domain-containing protein 2; TLN1: talin 1; TUBA4A: tubulin alpha 4A; HSPA8: heat shock protein family A (Hsp70) member 8; PLA2G10: phospholipase A2 group X; F13A1: coagulation factor XIII A chain; CFHR4: complement factor H related 4; PDGFA: platelet-derived growth factor subunit A; MUC1: mucin 1, cell surface associated; ITGAM: integrin subunit alpha M; CLU: clusterin; AHSG: alpha 2-HS glycoprotein; ECM1: extracellular matrix protein 1; FGB: fibrinogen beta chain; FGG: fibrinogen gamma chain; PD-L1: programmed death-ligand 1; THBS1: thrombospondin 1; ACPP: Acid Phosphatase 3; FASN: fatty acid synthase; FKBP5: FKBP prolyl isomerase 5; FAM129A: niban apoptosis regulator 1; FOLH1: folate hydrolase 1; KLK2: kallikrein related peptidase 2; KLK3/PSA: kallikrein related peptidase 3/prostate-specific antigen; MSMB: microseminoprotein beta; NKX3-1: NK3 homeobox 1; NDRG1: N-Myc downstream regulated 1; NEFH: neurofilament heavy chain; Rab27A: Ras-related protein Rab-27A; TGM4: transglutaminase 4; FLNA: filamin A; AMBP: alpha-1-microglobulin/bikunin precursor; CHMP4A: charged multivesicular body protein 4A; CHMP4C: charged multivesicular body protein 4C; FABP5: fatty acid binding protein 5; FOLR1: folate receptor alpha; Rab35: Ras-related protein Rab-35; RALB: Ras Like Proto-Oncogene B; TMPRSS2: transmembrane serine protease 2; TPP1: tripeptidyl peptidase 1; CEACAM-5: CEA cell adhesion molecule 5; EPS8L2: epidermal growth factor receptor kinase substrate 8-like protein 2; MSN: moesin; MUC1: mucin-1; TALDO1: transaldolase 1; CLU: Clusterin; C1R: complement C1r; APOA1: apolipoprotein A1; GAP43: growth associated protein 43; NRGN: neurogranin; SNAP25: synaptosome associated protein 25; SYT1: synaptotagmin 1; Aβ42: amyloid beta 42; SYNGR3: synaptogyrin-3; PSMA7: proteasome 20S aubunit alpha 7; CPNE3: copine 3; CD82: cluster of differentiation 82; ZIP4: zinc transporter ZIP4; CA125: cancer antigen 125; TDP-43: TAR DNA-binding protein 43, TGS101: tumor susceptibility 101.

## Data Availability

Not applicable.
